# A Web-Based Communication Platform to Improve Home Care Services in Norway (DigiHelse): Pilot Study

**DOI:** 10.2196/14780

**Published:** 2020-01-20

**Authors:** Linn Nathalie Støme, Tron Moger, Kristian Kidholm, Kari J Kværner

**Affiliations:** 1 Centre for Connected Care Oslo University Hospital Oslo Norway; 2 Institute for Health and Society University of Oslo Oslo Norway; 3 Centre for Innovative Medical Technology University of Odense Odense Denmark

**Keywords:** early health technology assessment, eHealth, primary care, innovation, behavioral data

## Abstract

**Background:**

Home care service in Norway is struggling to meet the increasing demand for health care under restricted budget constraints, although one-fourth of municipal budgets are dedicated to health services. The integration of Web-based technology in at-home care is expected to enhance communication and patient involvement, increase efficiency and reduce cost. DigiHelse is a Web-based platform designed to reinforce home care service in Norway and is currently undergoing a development process to meet the predefined needs of the country’s municipalities. Some of the main features of the platform are digital messages between residents and the home care service, highlighting information on planned and completed visits, the opportunity to cancel visits, and notifications for completed visits.

**Objective:**

This study aimed to test the usability and economic feasibility of adopting DigiHelse in four districts in Oslo by applying registry and behavioral data collected throughout a one-year pilot study. Early health technology assessment was used to estimate the potential future value of DigiHelse, including the predictive value of behavior data.

**Methods:**

Outcome measures identified by stakeholder insights and scenario drafting in the project’s concept phase were used to assess potential socioeconomic benefits. Aggregated data were collected to assess changes in health consumption at baseline, and then 15 and 52 weeks after DigiHelse was implemented. The present value calculation was updated with data from four intervention groups and one control group. A quasi-experimental difference-in-difference design was applied to estimate the causal effect. Descriptive behavioral data from the digital platform was applied to assess the usability of the platform.

**Results:**

Over the total study period (52 weeks), rates increased for all outcome estimates: the number of visits (rate ratio=1.04; *P*=.10), unnecessary trips (rate ratio=1.37; *P*=.26), and phone calls (rate ratio=1.24; *P*=.08). A significant gap was found between the estimated value of DigiHelse in the concept phase and after the one-year pilot. In the present pilot assessment, costs are expected to exceed potential savings by €67 million (US $75 million) over ten years, as compared to the corresponding concept estimates of a potential gain of €172.6 million (US $193.6 million). Interestingly, behavioral data from the digital platform revealed that only 3.55% (121/3405) of recipients actively used the platform after one year.

**Conclusions:**

Behavioral data provides a valuable source for assessing usability. In this pilot study, the low adoption rate may, at least in part, explain the inability of DigiHelse to perform as expected. This study points to an early assessment of behavioral data as an opportunity to identify inefficiencies and direct digital development. For DigiHelse, insight into why the recipients in Oslo have not made greater use of the Web-based platform seems to be the next step in ensuring the right improvement measures for the home care service.

## Introduction

The era of digital health and the demand for health information technology (HIT) brings enormous opportunities for both patients and professional users [1]. While HIT is the technology used in electronic health (eHealth) services, eHealth itself is defined as the interaction between medical informatics, public health, and businesses, referring to health services and information delivered or enhanced through the internet and related technologies [2]. One promise of eHealth solutions is that, through enhanced communication and patient involvement, and increased efficiency, reduced costs for the health care service may be achieved. It is also assumed that eHealth may enhance the quality of care by increasing transparency and availability between different health suppliers. There is, however, a discrepancy between the expected value of such interventions and the empirically demonstrated benefits [3,4]. There is a lack of case studies demonstrating the assumed cost-effectiveness and efficacy of eHealth solutions, and research to promote value-based health care in this field has been requested [3,5].

Web-based communication platforms are intended to enhance health in both somatic and mental health care [6-8]. Such platforms have shown success in reaching individuals who are hard to contact, in lifestyle behavior change, and the delivery of individualized online care [7,9,10]. For chronic illnesses, enabling people to administer their treatment and care may increase compliance to treatment regimens and improve quality of life. The translation of the Diabetes Prevention Program to online treatment is one such example [11]. The failure of adoption by end-users, however, is a challenge faced by these Web-based interventions. Accordingly, end-user engagement in the development of these interventions has been recognized as essential to increase adoption rates when they are introduced [12,13].

A health service characterized by efficiency and high quality can only be achieved if patient outcomes and costs of delivery are addressed [14]. When facing the complex health care system, not only do technical and legal issues appear, but so do organizational, economic, and social aspects [1]. User-centric design can be employed from the earliest exploratory stages to help understand and design for the needs, goals, limitations, capabilities, and preferences of all stakeholders [15]. Recommendations from an international workshop in the United Kingdom on how to create, evaluate, and implement effective eHealth interventions highlights new evaluative challenges in the field. Due to the swiftly changing technological landscape, these UK authors emphasized challenges such as continuous technological adaption and problems identifying valid outcome measures for assessment of costs and patient benefits [16]. Thus, to adjust to the rapidly changing context, standard methods for development and assessment will benefit from including the whole development cycle. Access to data and valid information from a conceptual stage of development may, however, be demanding, which could explain the lack of empirical evidence concerning the effect of eHealth interventions [3,17].

Health technology assessment (HTA) is traditionally used to provide decision support in the implementation phase of new or current health technology. HTA is defined as an interdisciplinary process for synthesizing information about medical, social, economic, and ethical issues related to the introduction of a new health technology [18]. To improve the pace and efficiency of the development and assessment of health innovation, new methods for early HTA are emerging in the literature [19]. Early HTA is a form of HTA that evaluates technologies still in development and can be defined as the initial examination of the medical, economic, social, and ethical implications of a health intervention to determine the potential of its incremental value in health care [20,21]. A standard model for early HTA is yet to be established, so research is needed to validate the proposed approaches to early HTA emerging in the literature [22].

DigiHelse is an intervention designed to reinforce the home care service in Norway and is currently undergoing a procurement process in the county’s municipalities. This is the second of a series of two studies reporting on the effects of implementing the Web-based communication platform, and the first study reported on the early assessment of potential socioeconomic value in the concept stage of the project. DigiHelse was designed and developed to integrate a national Web-based communication platform for recipients of home care services. The main features of the platform were digital messages between residents and the home care service, visualizing agreed upon and completed visits with their associated information, the option to cancel visits, and final notifications of completed visits. In the concept stage of development, data was collected from stakeholders and experts to build scenarios to show the potential value of the intervention. Based on the findings, the project was granted additional funding and proceeded to its pilot phase in four districts in Oslo. Throughout this pilot study, the project needed to collect evidence on its potential benefits to ease the procurement process in other municipalities in the country.

DigiHelse is an example of an eHealth intervention still in development; thus, there is an opportunity to perform assessments on the different stages of the development cycle. A stepwise decision process with several evaluation points and iterative adoptions of the solution has been incorporated in the implementation plan to ensure that the final solution meets the needs of the end-users. This study aimed to test the usability and economic feasibility of adopting DigiHelse in four districts in Oslo by applying registry and behavioral data collected throughout a one-year pilot. Early HTA was used to estimate the potential future value of DigiHelse, including the predictive value of behavior data.

## Methods

### Population

The target population for the intervention is composed of all the recipients of the home care service in Norway, their next of kin, and the service providers of the home care service. The home care service in Norway is a part of the country’s primary health care service. Norway has 426 municipalities that are responsible for the provision of services in primary care. Operations directed under primary care are typically health services provided outside an institution (with a preferred emphasis on health promotion and preventive work), general medical care (general practitioner ), and nursing services outside the hospital. Nurses and doctors in preventive and long-term care services are usually employed in municipal health care [23]. Although the municipalities in Norway dedicate a significant part of their budgets to health services (about one quarter), the home care service struggles to meet an increasing demand for health care under the constraints of a restricted budget [24]. During 2016, there were 355,635 unique recipients of nursing and care services nationally, which equates to 6.7% of the Norwegian population. Of the unique recipients of nursing and care services, 85% received home-based services, and about 2.7 million visits ware performed every week [25].

### The Intervention

This study was set in Oslo in 2018. The purpose of DigiHelse was to digitalize the dialogue between recipients and professionals in home care services in Norway through the development and implementation of a national Web-based platform. All recipients of home care services in four districts in Oslo were offered DigiHelse, in addition to regular services, in a one-year pilot project from autumn 2017 to the next year. The utilization of DigiHelse was completely voluntary. The project is based on the existing “Helsenorge.no” platform from the Norwegian Directorate of e-Health, which provides national digital health services. The realization of digital services in this project supports the overall objective of the development of information and communication technology in the health care sector to provide citizens with access to simple and secure digital services [26].

The intervention intends to cover the following objectives and needs:

- Support relatives who are involved in care tasks and strengthen the interaction between service providers and relatives through the possibility of secure digital dialogue and an overview of visits.
- Support service recipients in enhanced coping, safety, and involvement in their daily lives by providing an overview of visits and facilitating dialogue with the home care service, so that they can express their experiences and needs.
- Ensure that the home service can organize tasks more rationally and cooperate better with service recipients and relatives.
- Ensure that messages from relatives and recipients are captured and followed up with appropriately, such that phone inquiries are reduced, tasks can be registered at a more favorable time, and unnecessary trips to the recipient can be reduced.

### Choice of Health Outcomes

#### Summary

In the concept stage of DigiHelse, a multidisciplinary team of stakeholders managed to identify both quantitative and qualitative outcome measures comparing the new solution to the current situation. Through scenario building, a present value calculation on socioeconomic impact was carried out. The outcome measures, based on each scenario elaborated on in the previous study of DigiHelse, are presented below.

#### Increased Predictability for Recipients

Notifications of appointments and any delays might give recipients a greater sense of predictability and greater confidence in the home care services. Digital services may also provide better information security for recipients than email and texting, thus more thoroughly safeguarding the privacy of the recipients. In the concept assessment, increased predictability gave a predicted annual value of €408.4 million (US $458.3 million) per year. This was based on the assumption that if the recipients knew the exact arrival time of their home service care, an hour waiting time per visit might be saved. This effect was not included in the present value calculation as the value of free time is debatable.

#### Increased Involvement From Relatives and Volunteers

Improved communication between relatives and the home service was assumed to amount to savings of €13.8 million (US $15.5 million) a year. For relatively self-sufficient recipients, relatives and volunteers may carry out one visit per month on average.

#### Increased Predictability of the Home Care Service

The assumption in the concept stage was that the staff in the home care service might be able to better manage their workday by using digital channels rather than the telephone. They may experience reduced time consumption for administrative tasks and have more time for preventive work. Increased predictability of the home service may also result in fewer unnecessary trips to the users, as unwanted visits may be easily canceled in the portal. With the ability of the user to digitally cancel and postpone visits, a reduction of 30% of unnecessary trips was estimated, which results in assumed savings of €3.8 million (US $4.3 million) a year.

#### Greater Dialogue Efficiency and Time Management

Through interviews with professionals from the home services, the stakeholders estimated potential administrative time savings in administrative time of 30 minutes per day, with an hourly rate of €46 (US $51.60) with digital communication, resulting in savings of €7.1 million (US $8 million) a year.

#### Reduced Phone Inquiries

The estimated impact of reduced phone inquiries may amount to €1 million (US $1.2 million) per year on a national basis. To assess whether the intervention may reduce phone inquiries that otherwise could be solved digitally, the project group conducted a phone survey in Oslo and Bergen. After the survey, a scenario where digital communication could reduce phone inquiries to the home service by 40% was built.

#### Provide a Technical Basis for Developing Digital Services

Providing a technological basis for developing digital services may result in a one-time saving of €18.25 million (US $20.5 million). If 50% of the municipalities in Norway each procure a platform, they will, on average, consume €100,000 (US $110,000) each, including procurement, infrastructure, licenses/rent, etc. This effect was not included in the present value calculation because the digitalization of home services is still not statutory in the country’s municipalities.

In the present study, three outcomes (increased involvement from relatives and volunteers, increased predictability of the home care service, and reduced phone inquiries) were reassessed using empirical data from the one-year pilot in four districts in Oslo, and a control district. The remaining outcome measures will appear unchanged in the present value calculation, as will the unit costs of investment, training, and maintenance.

### Data Sources

In this pilot assessment, descriptive behavioral data from the Web-based platform was collected to study the usability of the platform. Data points, such as the number of digital users, digital inquiries, and active users, were retrieved from the platform’s server. In this study, we used behavioral data on the number of active users to study usability. All recipients in the intervention districts were offered the chance to log into the platform and create a profile. The number of active users is defined as the number of users who not only created a profile but also had interactions with the home care service in the platform. Aggregated data from the electronic patient record (EPR) system Gerica was retrieved to study changes in health consumption in the home care service in the four intervention districts and one control district in Oslo. Data collection was performed through three measurement points in time: at baseline (the week before the intervention), during the short period (15 weeks after the intervention), and over the total study period (52 weeks after the intervention). Data was collected on the number of visits of the home service to the recipient to assess if the intervention may give an incentive to increase involvement from relatives and volunteers in the care of recipients.

Further, to assess if the option to cancel unwanted trips in the portal may result in fewer unnecessary trips and increased predictability of the service, data was also collected on the number of unnecessary trips by the home care service to the recipient. An unnecessary trip is when the home service arrives at a recipient’s home for a planned visit, and the recipient does not answer the door. Finally, to study if digital dialogue may reduce the number of phone calls to the home service, phone calls to the service were registered during the three measurement points. Input variables on the cost of the present value calculation are shown in Multimedia Appendix 1.

### Data Analysis

A 10-year present value calculation model with a discount rate of 4% was used to estimate the potential value of the intervention. The potential value was first estimated every year, and by employing the cost of investment, training, and implementation pace, the overall value was calculated over ten years. The assumption of the 10-year life cycle is based on national recommendations from the Directorate for Financial Management [27]. The data from the intervention and control group was analyzed using the quasi-experimental difference-in-difference design to estimate the causal effect and to update the present value calculation. Such a design is typically used to estimate the effect of an intervention by comparing the changes in outcomes over time between a population exposed to the intervention (intervention group) and a population not exposed (control group) [28]. A Poisson regression analysis was used to fit the model, as the dependent variables are counts of events.

First, to test for an effect of the intervention, interaction models with dummy variables were used for the intervention and the period. To assess both the short-term and long-term effects, analyses were done separately for time points one week before the intervention versus 15 weeks after, and before intervention versus 52 weeks after. The number of those exposed to the intervention in the model corresponded to the number of home care recipients (user base) in each group because all recipients in the intervention group had, in principle, access to DigiHelse, and all analyses are based on aggregate data. The interaction coefficient between the intervention and the time period dummies indicates the effect of the intervention. Second, to assess the effect of the proportion of active users in the intervention districts, an interaction model with continuous-time and continuous rates of digital users was used in each district. Different rates of active digital users were then extrapolated to assess how this would influence the rates for visits, unnecessary trips, and phone calls, and thereby, the costs in the present value analysis. All calculations were done in kr and converted to euros based on the exchange rate from May 2018 (9.54) [29]. All analyses were performed in Stata 15.1 (StataCorp, College Station, Texas, United States) and Excel 2010 (Microsoft, Redmond, Washington, United States).

## Results

### Study Parameters

Table 1 and Table 2 show the demographic distribution and aggregate data from the EPR system Gerica for each of the intervention districts and the control district. District 2 has the highest percentage of active digital users. This district has a relatively high percentage of immigrants, but the lowest percentage of people under retirement age. The user base is the number of recipients of home care services in each district, and the digital users are the recipients who have logged in to the digital platform. The active digital users are the recipients who use the portal to actively administer their services and contact with the home care service. Finally, the demographic data shows the composition of people over retirement age and immigrants of the total population in each district.

Table 3 shows the rates for the number of visits, the number of unnecessary trips, and the number of phone calls extracted from the EPR system for every ten users. The rates in the intervention and control groups at baseline, after 15 weeks (short period), and after 52 weeks (total study period), with their associated percentage changes compared to baseline, are presented. Also presented are *P* values for whether the difference over time is significantly different between intervention and control, which corresponds to whether the intervention has an effect.

The intervention group had a 12% (8.32/69.33) higher rate for the number of visits at baseline (77.65) compared to the control group (69.33). After 15 weeks (short period), the rate for the number of visits in the control group increased by 7% (4.97/74.30). In the same period, the rate for the number of visits also increased in the intervention group by 6% (4.26/81.91; rate ratio=1.06; *P*=.59). In the total study period (after 52 weeks), the rate for the number of visits increased in the control group by 7% (5.05/74.38), but by 11% (8.77/86.42) in the intervention group (rate ratio=1.04; *P*=.10). Both unnecessary trips and phone calls had a lower rate at baseline in the intervention group (19%) compared to the control group (28%) at baseline. However, over time the rates were further reduced in the control group compared to the intervention group for both unnecessary trips and phone calls.

Over the 52 total weeks of the study period, unnecessary trips decreased in the control group by 33% (–0.21/0.42), and the rate for unnecessary trips reduced in the intervention group by 10% (–0.05/0.46). This is still less than in the control group, with a rate ratio of 1.37 *(P*=.26*)*. Phone calls were reduced in the control group by 2% (–0.05/2.66) and increased in the intervention group by 22% (0.42/2.36), by a rate ratio of 1.24 (*P*=.08*)*. In conclusion, all point estimates indicate that the intervention increases the rates for all outcomes, although none of the intervention effects were significant.

**Table 1:** Description of user base.

Users		District 1	District 2	District 3	District 4	Control
**User base, n**						
	Baseline	812	667	746	1073	590
	Short period	874	684	741	1064	607
	Long period	863	662	802	1078	600
**Digital users, n**						
	Baseline	0	0	0	0	0
	Short period	19	46	32	33	0
	Long period	442	269	382	351	0
**Active digital users, n**						
	Baseline	0	0	0	0	0
	Short period	7	15	23	21	0
	Long period	21	21	36	43	0

**Table 2:** Demographic data of user base.

District	Population, N	Over retirement age, n (%)	Immigrants, n (%)
1	57,000	6954 (12.2)	15,960 (28)
2	36,000	1836 (5.1)	12,600 (35)
3	49,200	5806 (11.8)	17,220 (35)
4	49,800	6823 (13.7)	8964 (18)
Control	51,400	2878 (5.6)	20,046 (39)

**Table 3:** Outcome rates in the intervention and control groups for every ten users.

Outcome		Baseline (week 0)	Short period after intervention (after 15 weeks)	Short period change, rate (%)	*P* value	Total study period (after 52 weeks)	Long-period change, rate (%)	*P* value
**Rate of visits**								
	Intervention	77.65	81.91	4.26 (6)	—	86.42	8.77 (11)	—
	Control	69.33	74.30	4.97 (7)	.59	74.38	5.05 (7)	.10
**Rate of unnecessary trips**								
	Intervention	0.51	0.41	–0.1 (–20)	—	0.46	–0.05 (–10)	—
	Control	0.63	0.48	–0.15 (–24)	.83	0.42	–0.21 (–33)	.26
**Rate of phone calls**								
	Intervention	1.94	1.81	–0.13 (–7)	—	2.36	0.42 (22)	—
	Control	2.71	2.64	–0.07 (–3)	.75	2.66	–0.05 (–2)	.08

### Incremental Costs and Outcomes

In the prior concept stage assessment of the project, a 90% adoption rate of the digital portal DigiHelse was assumed. Applying behavioral data made available from the platform’s server revealed that the adoption rate after the one-year pilot was not as expected. Only 3.55% (121/3405) of active users were registered in the data, which makes it a challenge to both predict whether the precision and the fit of the concept model were good and compare the present value calculation with and without empirical pilot data. As such, the present analysis may only show that the control district improved over time compared to the intervention districts and that the adoption rate of the intervention was considerably lower than expected. From the difference-in-difference analysis, a 37% (0.46/0.34) increase in the rate of unnecessary trips in the intervention group was found, but this was given the observed adoption rates of around 3.55% (121/3405). Using continuous-time and adoption rates in the model and extrapolation to 50% active digital users, the effect of the intervention would have been a 128-fold yearly increased rate of unnecessary trips. The same trend was found for the number of visits. When extrapolating for 50% of active digital users, the effect of the intervention would be a 1.04 times higher increase in the intervention group compared to the control group. Finally, if there were 50% active users, the effect of the intervention would be a 55-fold increase in the phone call rate.

When including the outputs from the difference-in-difference model comparing the intervention and control group into the present value calculation model from the concept stage, the estimated value of the intervention changes radically (see Multimedia Appendix 2). The net present value of the intervention after adding data form the pilot is reduced by €241.8 million (US $271.3 million) over ten years from the first assessment, resulting in a loss of €62.2 million (US $69.8 million) over ten years. Based on the present pilot assessment, costs are expected to exceed potential savings by €67 million (US $ 75.2 million) over ten years, compared to potential gains of €172.6 million (US $ 193.7 million) from the prior concept assessment.

## Discussion

### Primary Findings

Through a case of early HTA employing empirical data from a pilot study, the present study updated effect estimates made in the concept stage of the development of DigiHelse. Based on the present pilot assessment, costs are expected to exceed potential savings by €67 million (US $75.2 million) over ten years, compared to potential gains of €172.6 million (US $193.7 million) from the first assessment. After one year, only 3.55% (121/3405) of recipients used the platform actively. The prior socioeconomic analysis, conducted in the concept stage of DigiHelse, was based on stakeholder insight and scenario drafting. Collecting empirical data from the one-year pilot of DigiHelse, the present study evaluated the potential value of the intervention and assessed the precision of early HTA using stakeholder analysis and scenario drafting. Three of the outcome measures identified in the first study constituted the basis for the difference-in-difference analysis, and related costs were analyzed using a 10-year present value calculation with a rate of 4%. We found a significant gap between the estimated value in the concept stage of DigiHelse and the estimated value using empirical data from the one-year pilot.

This may indicate that early assessment using stakeholder insight and scenario drafting applied in the concept stage was less precise than expected. Another explanation may be, at least in part, suboptimal pilot implementation, as it is known that adoption and diffusion of eHealth solutions may be time-consuming and require significant adaptation of work practices [30]. However, by assessing behavioral data on the actual use of the platform, an important issue likely to affect the outcome of the assessment was found: a very low rate of DigiHelse users among recipients of home care services. This may explain why there was no significant change in the outcome measures between the control and the intervention districts after the pilot.

A review study highlighting methodological challenges in early HTA emphasizes both the lack of proof on the efficacy of the methods and the absence of a standardized framework for early assessments [20]. Empirical and theoretical attempts have been made to fill the evidence gaps in early assessment modeling, with theoretical recommendations on the use of sophisticated mathematical techniques such as Bayesian modeling or Markov modeling [31-34]. Empirical models based on scenario drafting and expert elicitation have also been used to compensate for the lack of data and steer the innovation in the right direction [31,35-38].

Findings from the review showed how stakeholder insights and scenario drafting might be used in an early phase to collect data on patient outcomes and effects on costs [17,39,40]. However, there are some presented concerns are, such as the high uncertainty regarding the availability of adequate data sources for modeling outcomes and that the models suffer from the precision required for data input [31,41]. Although a strength of the present study was the availability of concept stage assumptions and assessment based on stakeholder insights and scenario drafting when empirical data from the present pilot were analyzed, lack of precision was found. In line with similar research on the subject, we found that early-stage analyses may suffer from loss of information, as they are unable to reflect all possible outcomes [39,42]. Further, it cannot be excluded that stakeholders may be positively biased towards the value of the technology in which they have a particular interest [43]. This may explain the identified gap between the estimated socioeconomic value and the value assessment based on empirical pilot data in the present study.

While the low acceptability rate among recipients of the home care service in Oslo was an important concern found in the present study, other studies addressing the acceptance of eHealth solutions tested in clinical settings have indicated high patient acceptability rates [44-46]. However, it is unclear how the adoption rate of eHealth solutions may be affected once the technology is moved outside the boundaries of the clinic and is implemented in users’ homes. Discrepancies in access to the internet and technological literacy in different subgroups may influence the adoption rate and, thus, the estimated improvement in efficiency and cost reduction expected from the implementation of eHealth [47-49]. Identified subgroups that are especially challenged by eHealth solutions are the elderly [47,48], minorities [49], and the socioeconomically disadvantaged [48,49].

Effective adoption among users is a prerequisite for successful implementation, and the effectiveness of eHealth is compromised if the solutions are suboptimally implemented. Discomfort with the new technology and a preference for well-known, earlier provided services are reasons reported to influence the adoption of eHealth technologies [50,51]. Qualitative methods are needed to explore the experienced discomfort about or preference for existing analog health services. Such methods are increasingly being explored to accompany quantitative assessments of complex innovations to provide a deeper understanding of the adoption of eHealth [52]. While quantitative methods explore relationships between digitalization and disease outcomes, qualitative methods may provide a deeper understanding of contextual factors influencing these relationships, such as information on drivers and barriers to technological implementation [53].

The engagement of end-users in collaboration with product developers may succeed in increasing acceptability in particularly vulnerable groups by incorporating favorable eHealth designs to overcome barriers, although this may not be sufficient [54]. Predictive behavioral data represents another important tool, as it provides valuable information on the usability of a digitalized service and its corresponding population, and thus may determine whether the predefined intent of the new service is met. The digitalization of services in health care provides a new, potentially valuable data source as real-time data can be extracted and analyzed at any time [55]. According to the Lean Startup framework, behavioral data from initial testing provides essential information on how the market will respond to a service or a product [56]. Measuring quantifiable behavioral data outcomes provides an opportunity to assess usability [57]. Qualitative information on the directions of the developmental improvements of services may then be assembled from the same study sample. This allows for iterative modifications and adaptations at the initial project phase to avoid the implementation of ineffective services. Through this process, the likelihood of developing a user-centric service which complies with market expectations may increase as the early assessment of behavioral data provides the ability to test whether the service meets its initial intent and contributes to value-based health care [56].

### Limitations

There are several limitations of this study. Firstly, health economic analysis commonly presents results as cost per patient. The present analysis applied the net present value of the presented investment, weighting potential benefits against investment costs. In this case, the model was chosen due to the significant heterogeneity among recipients receiving home services and the early nature of the analysis.

Further, a quasi-experimental design was used. This means that many confounders may affect the results, such as changes over time independent of the intervention, aging in the population, and heterogeneity between the intervention and control groups. The homogeneity of the districts in the analysis may also be questioned due to the baseline data. To increase the representativeness of the selected control group, data could preferably have been collected from more than one control district. An increased number of measurement points before the intervention would have provided an opportunity to assess trend assumptions between the control and intervention group, which is crucial for difference-in-difference analyses.

Further, if the behavioral data had shown a higher adoption rate, both these issues would have been resolved before the difference-in-difference analysis. In addition to empirical results, the present value model could have been used to predict the socioeconomic outcomes if the adoption rate was 90%. However, given the unexpectedly low adoption rate, collecting more measurement points, and performing a sensitivity analysis of the findings was deemed futile. It should also be taken into consideration that the value of DigiHelse was calculated on a national basis, although, due to the Norwegian municipal health budget autonomy, it is uncertain whether all municipalities will implement the service. A final limitation of this study is that the analyses are based on aggregated numbers and not individual data. On the positive side, the database is larger than typical pilot studies; however, it comes with an inability to connect data sources to adjust for confounders on the individual level.

### Conclusion

Measuring objective behavioral data provides an important source to assess usability. This study reported on the attempt to evaluate methods for early HTA by reassessing DigiHelse by comparing pilot intervention data to a corresponding control group. In this pilot study, the low adoption rate may, at least in part, explain the inability of the DigiHelse pilot to perform as expected. This study points to an early assessment of behavioral data as an opportunity to identify inefficiencies and direct digital development. Implementing eHealth solutions is known to be challenging and time-consuming. To ensure adoption, effective diffusion strategies are needed, including user training programs. For DigiHelse, learning strategies may be targeted to increase adoption in the next phase.

The integration of behavioral data in early planning and assessment provides an opportunity to address implementation challenges and user adherence, where early HTA modeling has a purpose. For DigiHelse, insight into why the recipients in Oslo have not made greater use of the Web-based platform seems to be the next step in ensuring the right improvement measures for the home care service. We encourage more research on early HTA and the use of behavioral data in case studies as tools to empirically demonstrate eHealth intervention benefits.

## Appendix

Multimedia Appendix 1 Summary of cost units.

Multimedia Appendix 2 Summary of priced effects in the concept phase and after the pilot phase.

Multimedia Appendix 3 CONSORT‐EHEALTH checklist (V 1.6.1).

## Abbreviations

eHealth: electronic health
EPR: electronic patient record
HIT: health information technology
HTA: health technology assessment
