# Supplementary material for: A Web-Based Communication Platform to Improve Home Care Services in Norway (DigiHelse): Pilot Study
Source: JMIR Form Res. 2020 Jan 20;4(1):e14780. doi: 10.2196/14780 (PMC6997925; doi:10.2196/14780)
Supplement: Multimedia Appendix 1 [file formative_v4i1e14780_app1.docx]

APPENDIX 1

Table 1: Summary of cost units

| Summary unit costs |  | Comment | | | |
| --- | --- | --- | --- | --- | --- |
| Full time equivalents in hours a year | 1695 |  | | | |
| Hourly rate of the home service in Euro | 46 | Internal human resources, time spent on training in municipalities | | | |
| Hourly rate of consultancy in Euro | 107 | System development, planning and implementation costs | | | |
| Increase in the proportion of full-time equivalents for technical operation of the solution per operating unit | 10% | 10% the first two years, then 5% | | | |
| Training needs of new service staff, hours per employee | 3 |  | | | |
| Number of employees in need of training in the new service | 5 |  | | | |
| Training in basic electronic messaging, number of hours per employee | 15 |  | | | |
| Number of employees per operating unit / municipality, including operating supplier, receiving training in basic electronic messaging | 5 |  | | | |
| Average increase in annual license / maintenance cost to EMR per operating unit in Euro | 1 677 |  | | | |
| Number of full-time equivalents within the care service | 67 000 | Statistics Norway 2014 (134,000 employees). We have assumed that 50% need training | | | |
| Time spent on planning organizational changes | 225 | Hours per municipality | | | |
| Time spent on staff training / organizational changes in hours | 2 |  | | | |
| Time usage training of recipients in hours | 0,5 |  | | | |
| Number of active users | 89 000 | We have assumed that 50% of users need training. | | | |
| Other operating and maintenance costs at Norsk Helsenett, health authorities and 800HELSE in Euro a year | 262 055-817 610 | Increases in pace with the implementation | | | |
| Implementation pace in years | 5 | 365 ICT operating units | | | |
| Lifecycle in years: | 10 | The life cycle of professional systems is considerably longer than the standard life of ICT equipment | | | |
| Implementation pace | **2018** | **2019** | **2020** | **2021** | **2022** |
| Number of operating units (cost driver) | 3 | 26 | 91 | 243 | 365 |
| Share of population (effect driver) | *18 %* | 42 % | 69 % | 90 % | 100 % |

Table 1 shows the input variables on the cost side of the present value calculation investment, the expected implementation pace, number of full-time equivalents and average unit costs for investments in digital infrastructure, training and technical support was based on national statistics (Statistics Norway).
