# Supplementary material for: A Web-Based Communication Platform to Improve Home Care Services in Norway (DigiHelse): Pilot Study
Source: JMIR Form Res. 2020 Jan 20;4(1):e14780. doi: 10.2196/14780 (PMC6997925; doi:10.2196/14780)
Supplement: Multimedia Appendix 2 [file formative_v4i1e14780_app2.docx]

APPENDIX 2

An increase in unnecessary tips, number of visits and number of phone calls were found when the intervention group was compared to the control group trend. The column for the early assessment in the concept stage (CS) is based on stakeholder analysis and the pilot stage column (PS) is built on the analysis with empirical pilot data. Leaving the three outcome measures Increased predictability for recipients, Greater efficiency with dialogue with citizens and better time management, and Provide technical basis for the development of digital services from the concept analysis unchanged. Table 1 shows how the effect of adding empirical data to the remaining three outcome measures Increased involvement from relatives and volunteers, Increased predictability for the home care service and Reduce phone inquiries reduced the potential value of the estimation such that the return of investment becomes negative. The column Difference (CS-PS) shows the incremental change in estimated value between the model from the concept stage and the pilot. The net present value of the intervention after adding data form the pilot is reduced by 241.8 million Euro over 10 years form the first assessment, resulting in a loss of 62.2 million Euro over 10 years. Due to the low adoption rate showed above, a sensitivity analysis was not carried out.

Table 1. Summary of priced effects in the concept phase and after the pilot phase

| Priced effect measures | Euro | Early assessment in a concept stage of innovation (CS) | Early assessment after the one year pilot (PS) | Difference  (CS-PS) |
| --- | --- | --- | --- | --- |
| **For residents** |  |  |  |  |
| Increased predictability for recipients | Mill. Euro. pr. year | (408,4) | (408,4) | 0 |
| Increased involvement from relatives and volunteers | Mill. Euro. pr. year | 13.8 | -7 | -20.8 |
| **For the home care service** |  |  |  |  |
| Increased predictability for the home care service | Mill. Euro. pr. year | 3.8 | -4.7 | -8.5 |
| More effective of dialogue with citizens and better time management | Mill. Euro. pr. year | (7.1) | (7.1) | 0 |
| Reduce phone inquiries | Mill. Euro. pr. year | 1 | -3.3 | -4.3 |
| Provide technical basis for the development of digital services | Mill. Euro. pr. year | (19.1) | (19.1) | 0 |
| **Total** | Mill. Euro. pr. year | 25.7 | -7.9 | -33.6 |
| **Net present value of the intervention** | Mill. Euro | 179.6 | -62.2 | -241.8 |
| **Present value investment cost in the public sector** | Mill. Euro | 5.5 | 5.5 | 0 |
| **Net present value per invested Euro in the public sector** | Euro | 3.2 | -1 | -4.2 |

Table 1 compares the results for the early assessment in the concept stage (CS) with the assessment performed with data from the pilot (PS) and the difference between the two (CS-PS). The table presents yearly estimates for the six outcome measures, net present value, present value investment costs and net present value per invested Euro in the public sector over 10 years. Values in parenthesis are not included in the present value calculation.
